# Image Feature Types and Their Predictions of Aesthetic Preference and Naturalness

**DOI:** 10.3389/fpsyg.2017.00632

**Published:** 2017-04-28

**Authors:** Frank F. Ibarra, Omid Kardan, MaryCarol R. Hunter, Hiroki P. Kotabe, Francisco A. C. Meyer, Marc G. Berman

**Affiliations:** ^1^Department of Psychology, University of ChicagoChicago, IL, USA; ^2^School of Natural Resources and Environment, University of MichiganAnn Arbor, MI, USA; ^3^Department of Psychological Sciences, Vanderbilt UniversityNashville, TN, USA

**Keywords:** aesthetic preference, naturalness, nature restoration, semantic cognition, visual perception

## Abstract

Previous research has investigated ways to quantify visual information of a scene in terms of a visual processing hierarchy, i.e., making sense of visual environment by segmentation and integration of elementary sensory input. Guided by this research, studies have developed categories for low-level visual features (e.g., edges, colors), high-level visual features (scene-level entities that convey semantic information such as objects), and how models of those features predict aesthetic preference and naturalness. For example, in Kardan et al. (2015a), 52 participants provided aesthetic preference and naturalness ratings, which are used in the current study, for 307 images of mixed natural and urban content. Kardan et al. (2015a) then developed a model using low-level features to predict aesthetic preference and naturalness and could do so with high accuracy. What has yet to be explored is the ability of higher-level visual features (e.g., horizon line position relative to viewer, geometry of building distribution relative to visual access) to predict aesthetic preference and naturalness of scenes, and whether higher-level features mediate some of the association between the low-level features and aesthetic preference or naturalness. In this study we investigated these relationships and found that low- and high- level features explain 68.4% of the variance in aesthetic preference ratings and 88.7% of the variance in naturalness ratings. Additionally, several high-level features mediated the relationship between the low-level visual features and aaesthetic preference. In a multiple mediation analysis, the high-level feature mediators accounted for over 50% of the variance in predicting aesthetic preference. These results show that high-level visual features play a prominent role predicting aesthetic preference, but do not completely eliminate the predictive power of the low-level visual features. These strong predictors provide powerful insights for future research relating to landscape and urban design with the aim of maximizing subjective well-being, which could lead to improved health outcomes on a larger scale.

## Introduction

Previous research on affective and aesthetic responses to environments and visual stimuli has had an important role in advancing our understanding of human interactions with the natural environment (Kaplan et al., 1972; Ulrich, 1983; Staats et al., 2010; Kardan et al., 2015a). Extending those advancements adds to the understanding of beneficial effects natural environments have on mood and cognitive performance (Berto, 2005; Berman et al., 2008; Kaplan and Berman, 2010; Dadvand et al., 2015), and health in a broad sense (for example empirical studies such as Ulrich, 1984; Cimprich and Ronis, 2003; Mitchell and Popham, 2008; Kardan et al., 2015b; see Hartig et al., 2014 for a review).

The restorative potential of nature has been theoretically and empirically linked with a strong affinity and aesthetic preference for natural environments (Wilson, 1984; Purcell et al., 2001; Van den Berg et al., 2003; Hartig and Staats, 2006; Han, 2010). Attention Restoration Theory (ART, Kaplan, 1995; Kaplan and Berman, 2010), along with other theories (Ulrich et al., 1991; Mayer et al., 2008; Valtchanov and Ellard, 2015), has been proposed to explain the relationship between nature and improved cognitive performance. For example, the Valtchanov and Ellard (2015) study showed, through measurements of eye movements and blink rates, that natural environments foster lower cognitive load than urban environments. One of the explanations provided by ART is that natural environments capture more bottom-up/involuntary attentional processes compared to urban environments, and that urban environments capture more top-down/directed attentional processes (Kaplan and Berman, 2010). ART hypothesizes that directed attention is required for executive functioning and self-control, thus, interactions with urban environments may worsen cognitive performance by taxing directed attention (Kaplan and Berman, 2010).

An important question then arises: what features constitute a *perceived* natural environment? If there are well-known benefits of natural environments on cognitive performances, mood and health outcomes, then understanding the features that constitute “nature” and make it preferable can be a powerful guide for informing research in landscape and urban design in order to subtly improve cognitive performances, mood and health outcomes on a large scale.

Berman et al. (2014) have approached the first part of this question (what constitutes nature) using computational techniques to decompose scenes into their low-level visual features (i.e., basic physical spatial and color features). In the Berman et al. (2014) study, participants rated images based on perceived naturalness, and found that several of the low-level visual features significantly correlated with naturalness ratings. In a related study, Kardan et al. (2015a) used the same 307 images as in the Berman et al. (2014) study and participants rated the images for preference. Kardan et al. (2015a) then used the low-level features as independent variables in a model predicting preference. Further, Kardan et al. (2015a) reported that naturalness variance of a scene that was not modeled by the low-level visual features, was highly associated with the aesthetic preference for the scene, suggesting that there could be higher level semantic content in natural scenes that make them more preferable compared to man-made scenes. These studies provided insight into the way scenes could be interpreted in terms of their low-level features and how those features may be used to make broader semantic judgments of naturalness and aesthetic preference.

Hunter and Askarinejad (2015) examined this idea from their design perspective, and took a multidisciplinary approach to select higher-level semantic features representing a continuum of natural to manmade environmental scenes. These researchers appealed to theories from environmental psychology, evolution/ecology, and design/aesthetics, to derive 62 high-level such features. Broadly speaking, high-level visual features (Hunter and Askarinejad, 2015) are perceived objects that carry semantic information of a scene such as sky, water, building, etc. The features were selected based on their theorized applicability to the design of urban and green spaces to maximize aesthetic preference and cognitive restoration capacity of those spaces.

However, no research as of yet has examined whether these high-level features might also predict aesthetic preference and the perceived naturalness of scenes, or whether they mediate some of the association between the low-level features and aesthetic preference or perceived naturalness ratings. Relatedly, high-level features are composed of low-level visual information (that is, any image can be decomposed in terms of edge and color properties), and low-level features may also carry high-level semantic information about naturalness (Oliva and Torralba, 2006; Walther et al., 2009; Kotabe et al., 2016). By teasing apart the composition of images into low-level features and high-level features, and examining how these features explain variance in scene aesthetic preference and naturalness judgments, we aim to obtain useful insights related to the ways these features can be used to inform research on the design of urban environments and greenspaces.

### A development of previous studies

The current analysis pulls principally from results and data presented in original papers by Kardan et al. (2015a) and Hunter and Askarinejad (2015). The Kardan et al. (2015a) research focused on low-level features (as originally defined by Berman et al., 2014), where, in an experimental setting, 52 research participants (26 female, mean age = 21.1) provided aesthetic preference ratings, using a 7-point rating scale for 307 images (naturalness ratings were similarly obtained from Berman et al., 2014). Participant aesthetic preference ratings were then modeled using low-level visual features as predictors. It is important to note that in the current study, data from 260 of the original 307 images were used; 47 images were excluded because they were vintage or presented in portrait (vs. landscape) orientation. The Hunter and Askarinejad (2015) research focused on taking a theoretically driven approach to defining high-level semantic features. They did not, however, use these features to predict aesthetic preference and naturalness judgments. As such, these researchers provide a useful toolkit for operationalizing high-level sematic features, and in this study, we use those features to predict aesthetic preference and naturalness judgments for the first time.

### Low-level visual features

Research from Berman et al. (2014) quantified 10 low-level visual features of environmental scene images. These low-level features included spatial characteristics such as edge density, straight-edge density and entropy, and color characteristics such as hue, saturation, and brightness. Table 1 shows a complete list of these low-level features.

**Table 1:** **Color and edge low-level features quantified for aesthetic preference and naturalness models**.

**COLOR FEATURES**
Hue (avg)
Saturation (avg)
Brightness (avg)
SDhue (hue standard deviation)
SDsaturation (saturation standard deviation)
SDbrightness (brightness standard deviation)
**EDGE FEATURES**
Straight Edge Density
Disorganized Edge Ratio
Edge Density
Entropy

Berman et al. (2014) calculated the color features using MATLAB's image processing toolbox's built-in functions (MATLAB and Image Processing Toolbox Release 2012b, The MathWorks, Inc., Natick, Massachusetts, United States). Hue (dominant wave length in the image), saturation (ratio of hue to other wavelengths in the image), and brightness (an image's color intensity—visibly, it is the amount of darkness/lightness in an image) were calculated per pixel in each image, and those values were then averaged for each image to determine the image's average hue, saturation and brightness, respectively. Based on the same pixel values, the standard deviations for each color feature (SDhue, SDsaturation, SDbrightness) were also calculated, which quantified the amount of diversity of those features in each image.

For edge detection, Berman et al. (2014) used MATLAB's built-in “edge” function set to “canny.” Canny edge detection uses a five-stage algorithm (Canny, 1986) to filter noise and track strong and weak edges. The edge density ratio was calculated as the ratio of edge pixels to total pixels for each image. The pixels belonging to long straight edges were then distinguished from other edge pixels to quantify straight edge density, as well as the ratio of the non-straight edges to total edge content which was labeled as the disorganized edge ratio of each image (see Berman et al., 2014 for more details). Finally, gray-scale entropy was calculated from the histogram of gray-scale intensity values across 256 bins; the more uniform an image's grayscale distribution, the greater its entropy.

**Figure 1:**
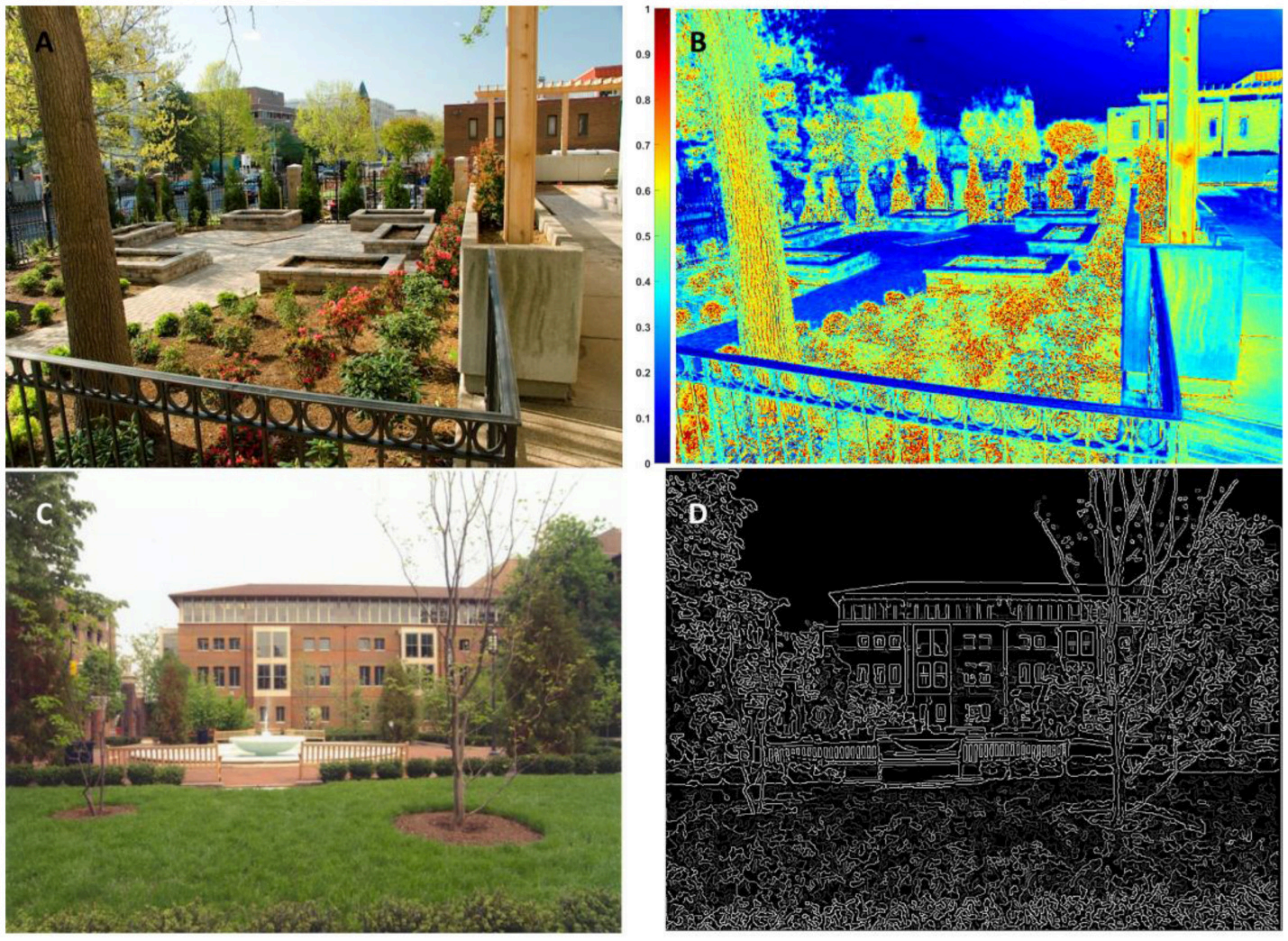
**(A)** First sample image **(B)** sample image's saturation map, pixels with hot colors have higher saturation **(C)** second sample image **(D)** edge density (ED) map of the sample image created from salient and faint edges of the image, detected using Canny edge detection.

Using the Berman et al. (2014) low-level feature algorithms, 307 images were decomposed by Kardan et al. (2015a). Figure 1 shows an example of edge feature detection, which was then quantified, and also shows an example of the decomposition of an image based on color saturation. Kardan et al. (2015a) then conducted a study in which these images were rated for aesthetic preference by participants using a 7-point Likert scale and the ratings were modeled using the low-level features as predictors.

### High-level visual features

Hunter and Askarinejad (2015) translational work reviewed scene preference predictions from 10 theories and identified 10 structure-content properties based on the investigated theories to predict scene preference. Supplementary Table 1 provides a breakdown of the investigated theories and the structure-content properties they embody that Hunter and Askarinejad (2015) theorized predict preference. Based on the 10 structure-content properties, the authors then derived 62 measurable high-level semantic features that can be used to predict scene aesthetic preference. See Supplementary Table 2 for a listing and definition of each of the 62 high-level features.

**Figure 2:**
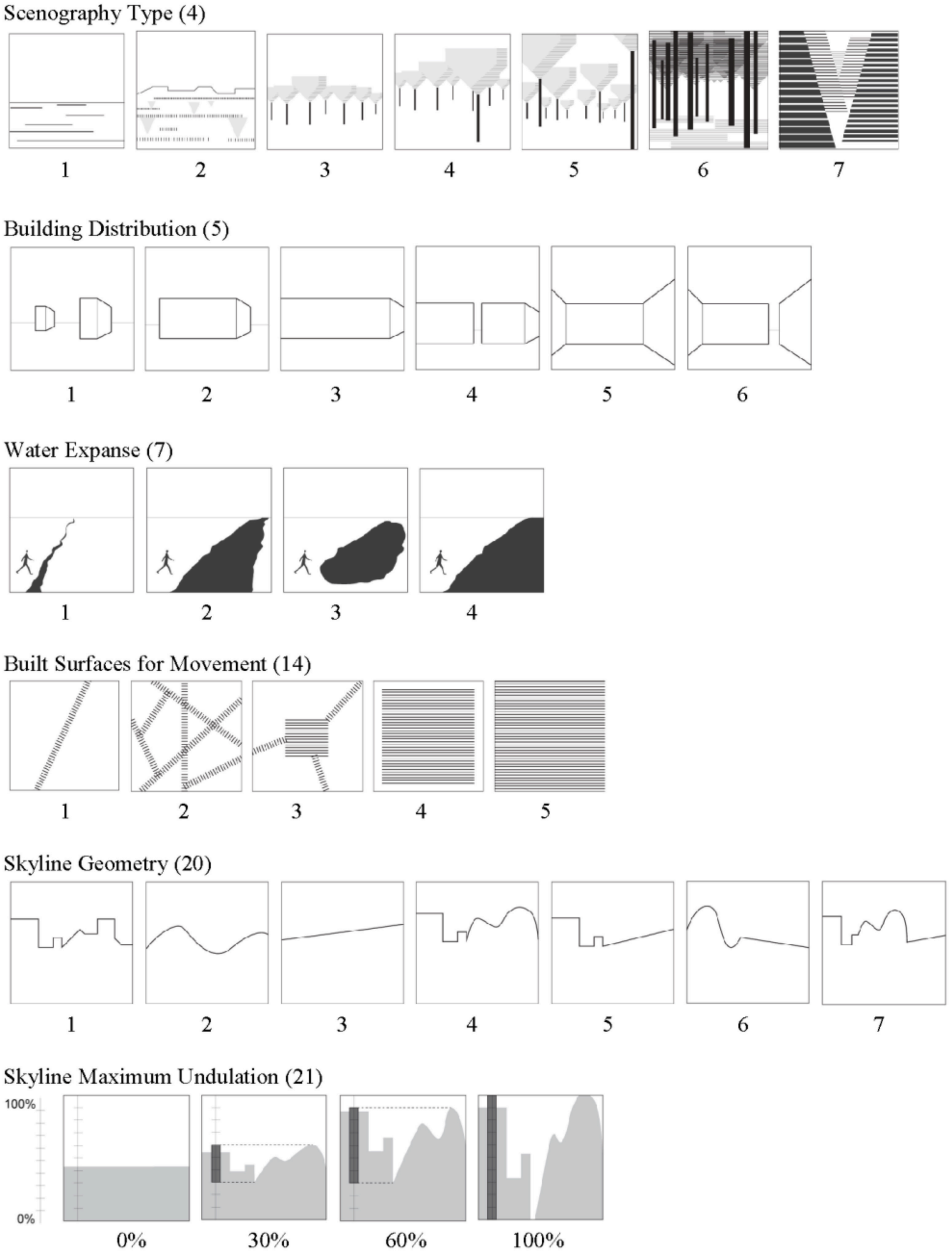
**High-level feature scoring diagram examples (6 of 62 high-level feature examples): Visualized definitions—character states of attributes with complex spatial definitions from Hunter and Askarinejad (**2015**)**. See Supplementary Table 2 for descriptions of character states depicted by each graphic. Parenthetical number = feature number listed on Supplementary Table 2.

To provide some examples of the various high-level features, we provide brief descriptions of 6 of the 62 high-level features; these six were selected as meaningful examples because they turned out to be predictive of aesthetic preference and/or naturalness in our statistical models. Those features are: Scenography Type, Building Distribution, Water Expanse, Built Surfaces for Movement, Skyline Geometry, and Skyline Maximum Undulation. All but Skyline Maximum Undulation are nominal variables. Figure 2 shows the pictorial abstractions used to assist scoring of these six high-level features. The Scenography Type describes the proximity of a viewer to the landscape beyond in terms of its sculptural form and scene depth; its character states represent a change from a more to a less expansive view. Building Distribution signifies the configuration of buildings or building clusters as they influence a viewer's visual and physical access to what is beyond. Water Expanse is based on physical or visual access across a water body in terms of movement by foot and ability to see the water boundary (e.g., not crossable and water boundary is/is not in sight). Built Surfaces for Movement is defined by the physical configuration of the designated circulation system found in the scene; character states describe the ability of paths to direct and orient. Skyline Geometry categorizes the composite shape of a scene's skyline based on the type of forms included; for example, buildings can add sharp corners or straight lines to the skyline while trees can add curves or straight lines depending on the distance between viewer and skyline objects. Finally, Skyline Maximum Undulation quantifies the maximum amount of vertical shift in the skyline based on distance between the highest and lowest points of the skyline; it is reported as percentage of the vertical frame height.

In this study we combine the work of Kardan et al. (2015a) and Hunter and Askarinejad (2015) to investigate the ways different image feature types predict aesthetic preference and perceived naturalness in conjunction with each other. This question is timely and important, given the demonstrable salubrious effects of exposure to natural environments and that little is known about what feeds into naturalness perceptions in the first place.

## Methods

### Image scoring and feature selection

All of the low-level features from the Kardan et al. (2015a) study were used in the present research. One change was that for this analysis hue of pixels in an image were aggregated as a circular scale (circular mean and circular standard deviation; Circular Statistics Toolbox for MATLAB; see www.kyb.mpg.de/~berens/circStat.html), rather than linear, which is more appropriate for calculating statistics of directional values (See Kardan et al., 2016 for an example).

The first major step in conducting our analysis was to score each image in terms of their high-level features. Some of the high-level features are defined as continuous variables, while others are defined as nominal variables (please refer to the Supplementary materials for descriptions of how each feature is defined and scored). In general, features operationalized as continuous variables were scored in terms of area or length, and measurements were made by creating overlay outlines in Photoshop with an interactive stylus pad; graphical data were then transferred to the Grasshopper plug-in for Rhinoceros 3D (a CAD application) to be processed by a program written for this analysis. To correct for different image sizes and shapes, data were interpreted in terms of percent of the image's total area, height or width, depending on which variable was involved. For the nominal features, images were visually evaluated by three trained scorers who worked independently and then compared results. Where discrepancies occurred, a 4th scorer was consulted followed by a group discussion for the final decision; the 4th scorer was used for 3% of the images. More details regarding other aspects of the scoring of high-level features can be found in Hunter and Askarinejad (2015). Eight high-level variables were excluded prior to analysis. Generally, these variables carried redundant information, were uninformative or were not applicable to our image set. The “Nature” and “Manmade” features were excluded from this analysis because these features are conglomerates of several other features and did not convey specific, meaningful semantic information concerning the role of high-level semantic features. For example, the “Nature” variable is created by adding together: “Non-veiling Vegetation” + “Sky Total” + “Water Total” + “Earth Total.” For the purposes of exploring high-level features, this type of conglomerate did not provide any unique information. Additionally, “Circulation Boundary Type' was excluded because it includes nominal levels describing the edge condition of circular surfaces and can include multiple levels of the nominal variable simultaneously (that is, one scene image can have multiple values on this dimension). Relatedly, the “Natural Phenomenon” and “Focal Objects” variables were also excluded as they allow for free writing of an infinite number of natural phenomenon and focal objects. Lastly, “Other” features were removed for similar reasons, namely, since “Other” is undefined in the scoring mechanism so the results were uninterpretable. Accounting for these preliminary exclusions, 54 of 62 high-level features remained at the onset of the statistical analysis.

### Model selection and data analysis

The purpose of our research was to investigate the ways low-level visual features relate to high-level visual features when predicting aesthetic preference as well as judged naturalness of the scene images. We conducted a new analysis of low-level features presented in the Kardan et al. (2015a) study incorporating the two changes mentioned previously: use of circular hue and omission of 47 images (recall these images were omitted for all analyses). Following that analysis we conducted similar analyses with only the high-level features to see how they predicted aesthetic preference and naturalness of the scenes. The reason for conducting analyses on just the high-level features without including the low-level features was to provide, for the first time, a set of analyses of high-level features analogous to those performed by Kardan et al. (2015a) on low-level features.

The high-level features were analyzed separately depending on whether or not they were scored as continuous or nominal variables. Those analyzed as continuous variables dealt primarily with the general semantic category of a scene, for example the percentage of total built surfaces, which quantified a general semantic category of how much *building* was present. The same can be said for sky, water, earth, and vegetation. In this way, we determined that the continuous variables represented by the high-level features could be thought of as measurements of a general semantic category en masse, and we placed them in linear models predicting aesthetic preference and naturalness.

In contrast, the high-level features scored as nominal variables breakdown these mass semantic categories into features of their design. For example, the Water Form nominal variable contains four levels within the general semantic category of *water*, but the nominal levels are particular to the *design* layout of water in the image and not to a continuum percentage measurement (Level 1 = installed water with engineered edges and a stylized aesthetic; Level 2 = installed water with engineered edges and a natural aesthetic; Level 3 = natural water body with engineered edges; Level 4 = natural water body with natural edges). All of the nominal variables were placed into general linear models (GLMs) predicting aesthetic preference and naturalness.

**Figure 3:**
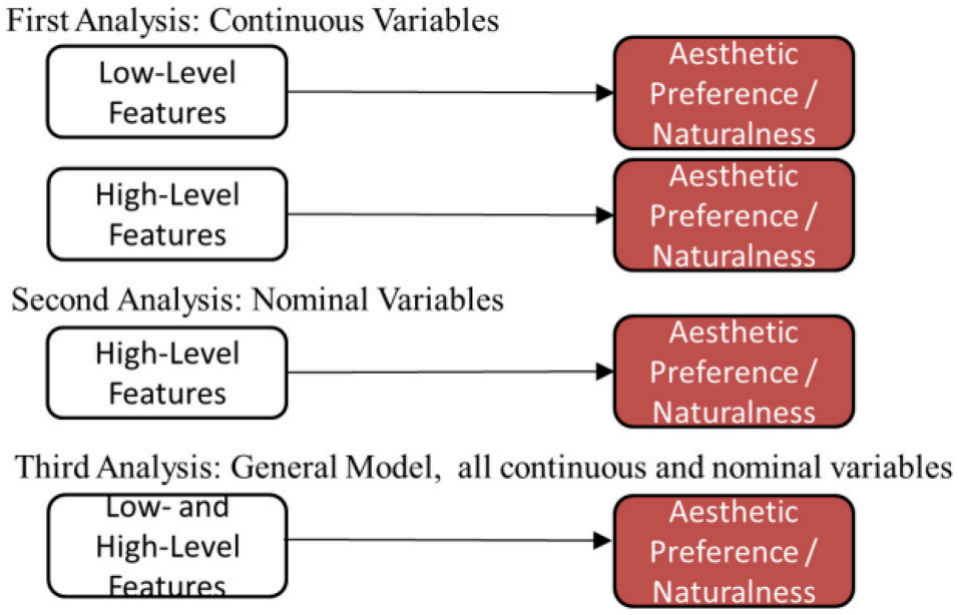
**Diagrams summarizing main analyses**.

We then modeled aesthetic preference and naturalness using both low-level and high-level features (continuous and nominal variables) together in a general linear model, and also developed mediation models demonstrating the ways high-level features mediate low-level features in predicting aesthetic preference (see Figure 3 for a summary of the 3 analyses).

During these analyses, we excluded variables to reduce the number of modeled predictors. Continuous variables were excluded using stepwise regression models, and variables entered into the GLMs that did not make significant contributions (*p* < 0.05) to the modeled variance of either dependent measure were excluded, and the GLM was re-computed until all predictors were significant. Of the total 72 low- and high-level features, 62 features were excluded from final aesthetic preference model, and 63 from the naturalness model.

Lastly, we investigated whether the high-level features mediated some or all of the effect of the low-level visual features when predicting aesthetic preference/naturalness of the scenes. Taking a hierarchical perspective of visual perception, low-level visual features have primacy in visual processing of a scene, which determines directionality of our mediation model; namely, the investigated mediators are high-level visual features, and not low-level visual features. From this perspective, note that high-level features are *composed* of low-level visual features; edges and colors are the basic components of a scene, and through grouping and segmentation, one can localize and identify high-level features. Several seminal models of visual perception propose such a sequence (e.g., Treisman and Gelade, 1980; Marr, 1982; Biederman, 1987), thus justifying the use of low-level visual features as the independent variable in our mediation models.

**Figure 4:**
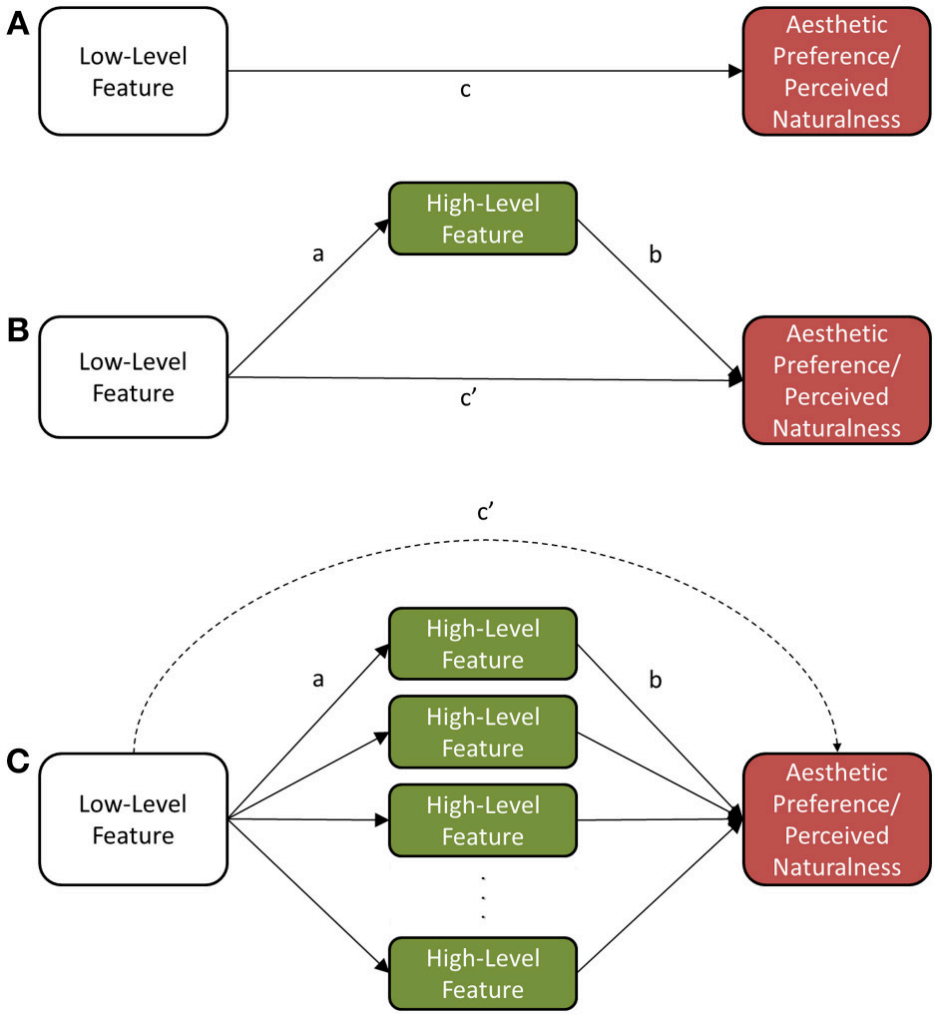
**Mediation models. (A)** Diagram of Total Effect, path c (no mediators). **(B)** Diagram of mediation model with one mediator, note direct effect path c′ and indirect effect path a^*^b which is equivalent to c-c′. **(C)** Diagram of multiple mediation model.

To begin our investigation, we correlated low-level features with aesthetic preference/naturalness, and selected those with a high association (*R*^2^ of at least 2%, see Cohen et al., 2013) with aesthetic preference/naturalness to investigate whether their effects were mediated by the high-level features (Figure 4A). Next, to select our mediators we correlated high-level features with aesthetic preference/naturalness and those with an *R*^2^ of at least 2% or above were selected as mediators. The selected variables were then placed into several simple mediation models (using PROCESS Macro v2.15 for SPSS, see Hayes, 2013 for a description) for each combination of low-level feature as predictor and high-level feature as mediator (see Figure 4B for a pictorial representation). In a final analysis, we also computed multiple mediation models for each selected low-level visual feature with all selected high-level features as mediators (as shown in Figure 4C).

As a supplementary inspection, we also looked at the high-level features that remained in our final model and computed one-way ANOVAs for matched pairs of continuous and nominal semantic variables (e.g., amount of “Water Total” across the nominal variable “Water Form”). This analysis allowed us to see, for example, if there were differences in mean “Water Total” (i.e., a continuous variable) across levels of the “Water Form” nominal variable, to gain more understanding of representation of these variables within this set of images. Results from the one-way ANOVA and plots can be found in Supplementary Figure 1.

## Results

### Continuous predictors: low-level and high-level features

All ten low-level features (see Table 1) were placed into a stepwise linear model predicting aesthetic preference and naturalness, resulting in $R_{\text{adj}}^{2}$ = 0.284, *F*_(5, 254)_ = 21.593, *P* < 0.0001 for naturalness, and $R_{\text{adj}}^{2}$ = 0.410, *F*_(8, 251)_ = 23.544, *P* < 0.0001 for aesthetic preference. The continuous high-level features explained more variance in aesthetic preference and naturalness, $R_{\text{adj}}^{2}$ = 0.529, *F*_(7, 252)_ = 16.963, *P* < 0.0001 for aesthetic preference and $R_{\text{adj}}^{2}$ = 0.843, *F*_(10, 249)_ = 139.843, *P* < 0.0001 for naturalness.

From the stepwise analysis, 5 of 10 low-level features and 7 of 26 high-level features remained in the model predicting aesthetic preference and 8 of 10 low-level features and 10 of 26 high-level features remained in the model when predicting naturalness. See Tables 2–5 for details of each model. High-level features explained more of the variance in naturalness than the low-level features in this analysis, which may not be too surprising considering that the high-level semantics are composed of the low-level visual features (the mediation analyses described later quantify this relationship in finer detail).

**Table 2:** **Low-Level visual features as predictors of aesthetic preference**.

**Feature**	**Standardized coefficient**	**Unstandardized coefficient**	**SE**	***t*-value**	***p*-value**	**Lower CI**	**Upper CI**
Intercept		1.732	0.494	3.509	0.001	0.760	2.704
Disorganized Edge Ratio	0.390	2.016	0.342	5.889	0.000	1.342	2.690
Brightness	0.265	3.067	0.639	4.800	0.000	1.809	4.326
Saturation	0.282	2.289	0.475	4.814	0.000	1.353	3.225
Hue	0.248	0.172	0.041	4.198	0.000	0.091	0.252
Total edge density	−0.262	−10.085	2.665	−3.784	0.000	−15.334	−4.836

*$R_{adj}^{2}$ = 0.284, F_(5, 254)_ = 21.593, P < 0.0001*.

**Table 3:** **Low-Level visual features as predictors of naturalness**.

**Feature**	**Standardized coefficient**	**Unstandardized coefficient**	**SE**	***t*-value**	***p*-value**	**Lower CI**	**Upper CI**
Intercept		5.966	2.521	2.366	0.019	1.000	10.931
Disorganized edge ratio	0.369	3.661	0.625	5.853	0.000	2.429	4.893
Brightness	0.249	5.541	1.125	4.928	0.000	3.327	7.756
Hue	0.228	0.304	0.074	4.077	0.000	0.157	0.450
Entropy	−0.257	−1.682	0.351	−4.795	0.000	−2.373	−0.991
Saturation	0.213	3.318	0.967	3.432	0.001	1.414	5.222
SDBrightness	0.218	9.311	2.381	3.911	0.000	4.622	13.999
Total edge Density	0.223	16.493	5.323	3.098	0.002	6.009	26.976
SDSaturation	−0.132	−4.423	2.105	−2.101	0.037	−8.569	−0.278

*$R_{adj}^{2}$ = 0.410, F_(8, 251)_ = 23.544, P < 0.0001*.

**Table 4:** **High-Level continuous features as predictors of aesthetic preference**.

**Feature**	**Standardized coefficient**	**Unstandardized coefficient**	**SE**	***t*-value**	***p*-value**	**Lower CI**	**Upper CI**
Intercept		4.369	0.129	33.848	0.000	4.114	4.623
Water total	0.351	0.033	0.004	7.307	0.000	0.024	0.042
Built structures total	−0.227	−0.010	0.003	−4.094	0.000	−0.015	−0.005
Built ground total	−0.197	−0.015	0.003	−4.302	0.000	−0.022	−0.008
Skyline vibrancy A	0.351	0.009	0.002	4.220	0.000	0.005	0.014
Skyline vibrancy B	−0.180	−0.002	0.001	−2.149	0.033	−0.004	0.000
Vegetation canopy	0.158	0.008	0.003	2.983	0.003	0.003	0.013
Sky veiled	−0.139	−0.024	0.009	−2.710	0.007	−0.041	−0.006

*$R_{adj}^{2}$ = 0.529, F_(7, 252)_ = 16.963, P < 0.0001*.

**Table 5:** **High-Level continuous features as predictors of naturalness**.

**Feature**	**Standardized coefficient**	**Unstandardized coefficient**	**SE**	***t*-value**	***p*-value**	**Lower CI**	**Upper CI**
Intercept		4.419	0.315	14.030	0.000	3.799	5.039
Built structures total	−0.401	−0.035	0.004	−8.845	0.000	−0.043	−0.027
Built ground total	−0.810	−0.119	0.024	−4.904	0.000	−0.166	−0.071
Water total	0.236	0.043	0.006	7.004	0.000	0.031	0.055
Skyline maximum undulation	−0.144	−0.018	0.004	−4.744	0.000	−0.025	−0.010
Skyline vibrancy A	0.252	0.013	0.002	5.191	0.000	0.008	0.018
Vegetation groundcover	−0.114	−0.012	0.003	−3.588	0.000	−0.018	−0.005
Non-veiling vegetation	0.181	0.013	0.003	3.652	0.000	0.006	0.019
Skyline vibrancy B	−0.166	−0.004	0.001	−3.463	0.001	−0.006	−0.002
Horizon line position	0.065	0.006	0.003	2.308	0.022	0.001	0.012
Built ground open	0.469	0.070	0.025	2.819	0.005	0.021	0.120

*$R_{adj}^{2}$ = 0.843, F_(10, 249)_ = 139.843, P < 0.0001*.

As a final investigation of the continuous variables, we placed all remaining low-level features and high-level features in a stepwise regression resulting in $R_{\text{adj}}^{2}$ = 0.596, *F*_(9, 250)_ = 43.507, *P* < 0.0001 for aesthetic preference, and $R_{\text{adj}}^{2}$ = 0.853, *F*_(12, 247)_ = 126.578, *P* < 0.0001 for naturalness. To note, from Tables 6, 7 (for both models) features involving *building* take on negative coefficient values, and features involving *water* take on positive coefficient values. These values demonstrate, that for increases of both preference and naturalness ratings, the amount of *building* decreases and the amount of *water* increases, which is consistent with what we might expect from the previously cited literature on preferences for natural versus urban environments.

**Table 6:** **Low- and High-Level continuous predictors of aesthetic preference**.

**Feature**	**Standardized coefficient**	**Unstandardized coefficient**	**SE**	***t*-value**	***p*-value**	**Lower CI**	**Upper CI**
Intercept		4.194	0.342	12.258	0.000	3.521	4.868
Water total	0.416	0.039	0.004	8.912	0.000	0.030	0.048
Built structures total	−0.156	−0.007	0.003	−2.797	0.006	−0.012	−0.002
Saturation	0.237	1.924	0.356	5.402	0.000	1.223	2.626
Hue	0.216	0.149	0.031	4.756	0.000	0.087	0.211
Skyline vibrancy A	0.382	0.010	0.002	4.912	0.000	0.006	0.014
Skyline vibrancy B	−0.266	−0.003	0.001	−3.729	0.000	−0.005	−0.002
Built ground total	−0.139	−0.011	0.004	−2.937	0.004	−0.018	−0.003
Total edge density	−0.150	−5.768	2.135	−2.701	0.007	−9.973	−1.562
Vegetation canopy	0.126	0.006	0.002	2.610	0.010	0.002	0.011

*$R_{adj}^{2}$ = 0.596, F_(9, 250)_ = 43.507, P < 0.0001*.

**Table 7:** **Low- and High-Level continuous predictors of naturalness**.

**Feature**	**Standardized coefficient**	**Unstandardized coefficient**	**SE**	***t*-value**	***p*-value**	**Lower CI**	**Upper CI**
Intercept		9.512	1.323	7.190	0.000	6.906	12.117
Built structures total	−0.400	−0.035	0.004	−9.149	0.000	−0.043	−0.028
Built ground total	−0.748	−0.110	0.023	−4.664	0.000	−0.156	−0.063
Water total	0.252	0.045	0.006	7.689	0.000	0.034	0.057
Skyline maximum undulation	−0.148	−0.018	0.004	−4.821	0.00	−0.026	−0.011
Entropy	−0.104	−0.681	0.172	−3.963	0.000	−1.019	−0.342
Hue	0.074	0.099	0.038	2.612	0.000	0.024	0.174
Built ground open	0.410	0.062	0.024	2.547	0.011	0.014	0.109
Skyline vibrancy A	0.205	0.010	0.002	4.210	0.000	0.006	0.015
Skyline vibrancy B	−0.132	−0.003	0.001	−2.751	0.006	−0.006	−0.001
Non-veiling vegetation	0.169	0.012	0.003	3.473	0.001	0.005	0.018
Vegetation groundcover	−0.108	−0.011	0.003	−3.534	0.000	−0.017	−0.005
Horizon line position	0.060	0.006	0.003	2.199	0.029	0.001	0.011

*$R_{adj}^{2}$ = 0.853, F_(12, 247)_ = 126.578, P < 0.0001*.

### Nominal predictors: design categories

Due to their stronger affiliation as design features rather than general semantic features, the nominal predictors are not as intuitive to understand as the continuous high-level semantic predictors, because each level of the nominal variables is representative of a different characteristic feature that do not operate on a continuum. For example, one level of the Water Expanse feature represents water features such as streams that are crossable and means of crossing them are visible in the image, versus a different level of Water Expanse that represents water features such as un-crossable bodies of water with no visible end, such as a large lake or ocean. Thus, to get a sense of how each nominal variable predicts aesthetic preference and naturalness, we ran separate general linear models for each nominal variable as a predictor for preference and naturalness. The results are shown in Supplementary Table 3. In sum, of the 27 nominal variables, when modeled individually, 21 significantly predicted aesthetic preference (*p* < 0.05) and 20 significantly predicted naturalness (*p* < 0.05). Notably, 8 variables that individually predicted aesthetic preference and 9 for naturalness have an $R_{\text{adj}}^{2}$ > 0.3. An impressive example of the predictive power of these nominal variables is that 65% of the variance in naturalness was explained by the Habitat Type Contextual variable alone (refer to Supplementary Table 2 for feature descriptions). These data led us to understand that despite the effective modeling of the continuous high-level features that are affiliated with general semantic information (e.g., amount of sky, water, earth, vegetation, or buildings), the nominal high-level features affiliated with more design qualities of a scene more strongly predict aesthetic preference and naturalness.

After placing all of the nominal variables in a general model to predict aesthetic preference and naturalness and reducing the model to only include significant contributors, 6 variables in the aesthetic preference model and 7 variables in the naturalness model remained and resulted in $R_{\text{adj}}^{2}$ = 0.572 for aesthetic preference and 0.868 for naturalness, compared to the continuous high-level variables at $R_{\text{adj}}^{2}$ = 0.529 for naturalness and 0.843 for aesthetic preference (in all cases *p* < 0.0001). The nominal variables are carrying slightly more predictive power than the continuous variables. See Table 8 for a list of predictors that remained in each model and their associated statistics.

**Table 8:** **Predictor estimates of preference and naturalness model with only significant nominal variables as predictors**.

**Feature**	**Degrees of freedom**	***F*-value**	***P*-value**
**PREFERENCE**			
Intercept	1	586.362	0.000
Scenography type	7	5.175	0.000
Building distribution	5	10.148	0.000
Habitat type contextual	7	3.388	0.002
Habitat type emulated	7	5.096	0.000
Viewer in shade	1	9.495	0.002
Lighting	1	11.923	0.001
**NATURALNESS**			
Intercept	1	494.821	0.000
Habitat type contextual	7	5.103	0.000
Habitat type emulated	7	8.781	0.000
Dominant cover type on circulation surfaces	8	5.701	0.000
Skyline geometry	7	15.463	0.000
Lighting	1	16.162	0.000
Windows	1	25.975	0.000
Vehicles	1	9.619	0.002

*F_(28, 231)_ = 13.343, $R_{adj}^{2}$ = 0.572, p < 0.0001*.

*F_(32, 227)_ = 54.094, $R_{adj}^{2}$ = 0.868, p < 0.0001*.

When we placed all remaining continuous and nominal variables together in models of aesthetic preference and naturalness, and reduced the number of modeled predictors by excluding non-significant variables, we produced a linear model for preference with an $R_{\text{adj}}^{2}$ = 0.684, *p* < 0.0001 (10 variables included) and for naturalness $R_{\text{adj}}^{2}$ = 0.887, *p* < 0.0001 (9 variables included). Table 9 provides a list of variables remaining in the model and their associated statistics.

**Table 9:** **Predictor estimates of full model**.

**Feature**	**Degrees of Freedom**	***F*-value**	***P*-value**
**AESTHETIC PREFERENCE**			
Intercept	1	386.886	0.000
Scenography type	7	6.601	0.000
Building distribution	5	6.284	0.000
Habitat type contextual	7	2.632	0.012
Habitat type emulated	7	3.340	0.002
Viewer in shade	1	8.950	0.003
Lighting	1	4.930	0.027
Water total	1	39.227	0.000
Built ground total	1	2.016	0.007
Hue	1	12.540	0.000
Saturation	1	14.267	0.000
**NATURALNESS**			
Intercept	1	332.222	0.000
Habitat type emulated	7	15.266	0.000
Habitat type contextual	7	4.509	0.000
Skyline geometry	7	6.102	0.000
Windows	1	11.256	0.001
Vehicles	1	23.322	0.000
Built structures total	1	47.907	0.000
Water total	1	13.075	0.000
Horizon line position	1	6.473	0.012
Hue	1	20.148	0.000

*F_(32, 227)_ = 18.536, $R_{adj}^{2}$ = 0.684, p < 0.0001*.

*F_(27, 232)_ = 76.187, $R_{adj}^{2}$ = 0.887, p < 0.0001*.

*Highlighted yellow are the high-level nominal variables, in green the high-level continuous variables, and in blue the low-level continuous variables*.

The next area of interest was to investigate the levels within each modeled nominal variable that had the largest and smallest mean values of aesthetic preference and naturalness. Now that we had determined the significant predictors for aesthetic preference and naturalness, we could tease apart the levels within these variables to understand the different ways each level was reflected in the outcome ratings. In this way, we could look toward a broader question of the design features associated with the modeled global semantic predictor that have large and small mean ratings to inform green/urban space design research. Table 10 provides a summary of these results. In this table we report the levels within the modeled nominal variables that carry the highest and lowest mean ratings (of the images containing these features) for aesthetic preference and naturalness (notice that from Table 8, variables Habitat Type Contextual and Habitat Type Emulated were significant predictors of both preference and naturalness).

**Table 10:** **Nominal variables predicting aesthetic preference and naturalness**.

**Nominal predictor**	**Level description (LX indicates level number)number)**	**Aesthetic preference**	**Mean naturalness**
Scenography type	(L1)Landscape extends from the viewer to a vista or a bird's eye view	5.623	NS
	(L5)Open area in the foreground with vegetation/objects that extend continuously into the distance	4.156	
Viewer in shade	(L1)Vantage point is shaded	4.907	NS
	(L0)Vantage point is not shaded	4.330	
Lighting	(L0)Manmade light source not present	4.318	NS
	(L1)Manmade light source present	2.403	
Building Distribution	(L0)No buildings	4.998	NS
	(L3)Building/clusters completely block the view beyond but a way to move beyond is inferred	3.6468	
Habitat type contextual	(L4)Coastal/edge area of a waterbody	5.548	5.516
	(L8)Urban/urbanized	3.940	2.684
Habitat type emulated	(L0)No built/designed emulation of a habitat type	5.134	5.793
	(L5)Savanna-like emulation	3.792	2.533
Windows	(L0)No windows present	NS	5.159
	(L1)Windows present		2.603
Skyline geometry	(L3)Shape of skyline is a straight line	NS	6.495
	(L1)Skyline shaped by sharp corners		1.796
Vehicles	(L0)No vehicles present	NS	4.249
	(L1)Vehicles present		2.566

*Level descriptions identify highest (in white) and lowest (in gray) mean ratings for images containing the modeled nominal variable. NS means that the nominal predictor was not a significant predictor of the corresponding dependent variable in the previous analyses (Table 8)*.

### Mediation analysis

After conducting correlational analysis between the low-level features and aesthetic preference, the selected predictor variables for our mediation analysis were: brightness, saturation, hue, disorganized edge ratio, straight edge density, and SD saturation. The various high-level mediators can be found in Table 11, as well as results of effect sizes from the mediation models (note: no significant mediators were found for SD Saturation, so this feature was excluded from analysis). Please see Supplementary Table 4 for mediation results for Naturalness.

**Table 11:** **Effect sizes from mediation analyses**.

Brightness^*^		**Straight edge density**		**Hue**		**Disorganized edge ratio**		**Saturation**	
**Total effect: 1.3642 95% CI:** −**0.0426, 2.771**		**Total effect:** −**7.7161 95% CI:** −**12.1559**, −**3.2763**		**Total effect: 0.1970 95% CI: 0.1157, 0.2783**		**Total effect: 1.8855 95% CI: 1.2962, 2.4748**		**Total effect: 2.4729 95% CI: 1.5256, 3.4202**	
**High-level feature**	**Direct effect (95% CI)**	**High-level feature**	**Direct effect (95% CI)**	**High level feature**	**Direct effect (95% CI)**	**High-level feature**	**Direct effect (95% CI)**	**High-level feature**	**Direct effect (95% CI)**
Skyline Vibrancy A	−0.011 (−1.3605, 1.3385)	Skyline vibrancy A	−3.4726 (−7.7891, 0.8439)	Sky open	0.2594 (0.1813, 0.3375)	Sky width in frame	2.1803 (1.6020, 2.7585)	Built structures open	1.4194 (0.5217, 2.3170)
Sky Open	−0.1709 (−1.7232, 1.3813)	Built structures veiled	−4.4413 (−8.9512, 0.0686)	Built structures open	0.099 (0.0214, 0.1766)	Built structures open	0.82391 (0.1864, 1.4614)	Built ground open	1.6518 (0.7219, 2.5818)
Water Open	−0.04 (−1.2646, 1.1845)	Sky open	−5.6367 (−10.0886, −1.1849)	Built ground open	0.1384 (0.0604, 0.2165)	Built ground open	1.0225 (0.3293, 1.7157)	Built structures total	1.4591 (0.5951, 2.3230)
Sky Total	0.3315 (−1.1676, 1.8305)	Built structures open	−2.1476 (−6.3448, 2.0496)	Sky Total	0.262 (0.1824, 0.3415)	Built structures total	0.7620 (0.1499, 1.3741)	Built ground total	1.6744 (0.7615, 2.5874)
Water Total	−0.0096 (−1.2104, 1.1913)	Built ground open	−3.2141 (−7.5843, 1.1560)	Built structures total	0.1007 (0.0259, 0.1755)	Built ground total	0.9771 (0.3016, 1.6527)		
Non-veiling vegetation	2.3335 (0.9314, 3.7356)	Built structures total	−0.6135 (−4.7989, 3.5719)	Built ground total	0.1363 (0.0591, 0.2136)				
Vegetation total	2.4569 (1.0129, 3.9009)	Built ground total	−2.8243 (−7.1729, 1.5243)						
Vegetation canopy	2.1953 (0.7858, 3.6048)								
**Multiple mediation models**									
**Brightness**		**Straight edge density**		**Hue**		**Disorganized edge ratio**		**Saturation**	
**Total effect: 1.3642 CI:** −**0.0426, 2.771**		**Total effect:** −**7.7161 CI:** −**12.1559**, −**3.2763**		**Total effect: 0.1970 CI: 0.1157, 0.2783**		**Total effect: 1.8855 CI: 1.2962, 2.4748**		**Total effect: 2.4729 CI: 1.5256, 3.4202**	
Direct Effect	−0.361 (−1.5274, 0.8056)	Direct effect	5.137 (1.1475, 9.1265)	Direct effect	0.1089 (0.0337, 0.1841)	Direct effect	0.2703 (−0.4369, 0.9775)	Direct effect	1.2608 (0.4045, 2.1171)

*All reported mediation analyses are only for the high-level features (mediators) with significant indirect effects after Holm-Bonferroni correction for 27 comparisons (Note SD Saturation is not included because it had no significant indirect effects). Multiple mediations computed using all significant mediators for the corresponding low-level feature. 5,000 bootstrap samples for bias corrected bootstrap confidence intervals. Color coding: green, full mediation; orange, partial mediation; blue, suppression*.

* *Brightness Total Effect was marginally significant (p = 0.057)*.

The total effect and direct effect results provide valuable information about the extent to which these low-level features are being mediated by the selected high-level features (see color coding in Table 11 indicating full and partial mediation, as well as suppression). Full mediation takes place when the direct effect of the predictor variable is no longer significant (confidence interval includes zero) with the introduction of the mediating variable, and partial mediation takes place when the direct effect of the predictor variable is still significant (non-zero confidence interval), but weakened when the mediator is introduced and the indirect effect is significant. Suppression occurs when the predictor variable becomes significantly stronger when the mediator is introduced in the model (MacKinnon et al., 2000). As Table 11 demonstrates, full mediation only takes place for Brightness and Straight Edge Density variables, Saturation is only partially mediated by the mediating variables, and the Built Structures Open feature mediates all of the tabled low-level features except Brightness. This brief survey highlights that there are different types of mediating relationships depending on which low-level visual features and high-level features are being modeled. To provide an example of how to interpret the total effect and direct effect values, see the multiple mediation results outlined in Table 11 for Disorganized Edge Ratio. For this multiple mediation, since the confidence interval for the direct effect (c′ = 0.2703) includes zero [−0.4369, 0.9775], the predictor variable is non-significant, i.e., fully mediated. The indirect effect (1.8855, −0.2703 = 1.6152 [0.9313, 2.4490]) is significant, and fully explains the relationship between Disorganized Edge Ratio and aesthetic preference. Also, it is important to note that all standard deviation low-level features representing variance in a given feature (i.e., SD Hue, SD Saturation, and SD Brightness) are absent, along with Entropy and Total Edge Density, so not all of the low-level features were selected to be in mediation models for aesthetic preference.

## Discussion

These results reveal that semantic information in image scenes, and in particular semantic information carried by design qualities, strongly predicts aesthetic preference and naturalness for images of scenes with mixed urban and natural content. To this end, we began by demonstrating that high-level features are stronger predictors than low-level features in predicting aesthetic preference and naturalness. Then, we showed that when comparing the two groups of high-level visual features, design features are stronger predictors of aesthetic preference and naturalness. Lastly, we showed that several of the effects of low-level visual features on aesthetic preference are mediated by high-level features, with evidence of full and partial mediation, as well as suppression. These results suggest that the role of low-level features in guiding aesthetic preference is complex and nuanced, with some low-level visual features affecting aesthetic preference through high-level semantics and other low-level visual features having more of a direct effect on aesthetic preference independent of higher-level semantics. As a general conclusion, we assert that not only is general semantic feature content important for predicting aesthetic preference and naturalness ratings, but also the semantic design formed by the featured content. As such, these modeled predictors should be used to inform greenspace and urban space design research.

**Figure 5:**
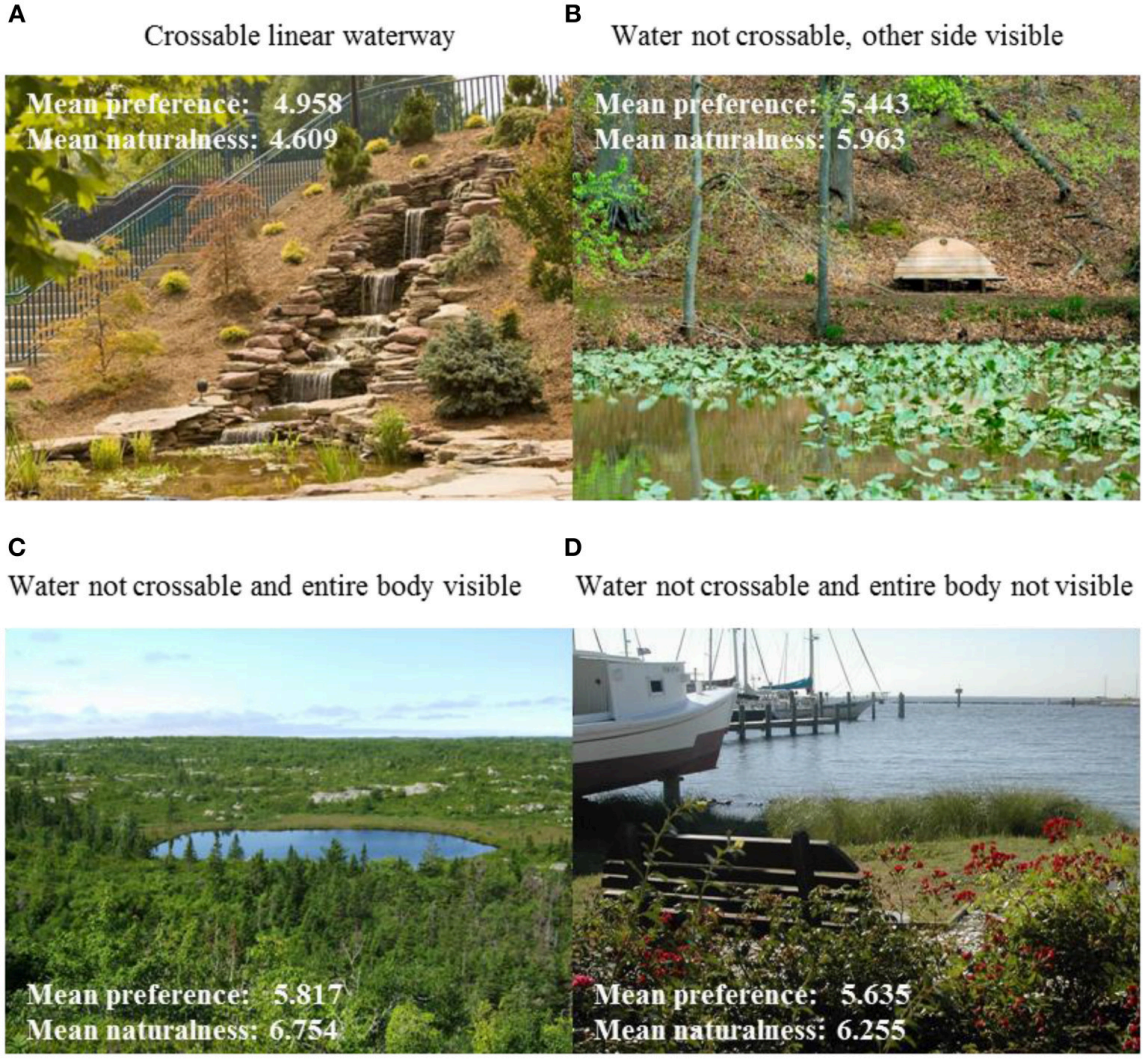
**Examples of images representing the four levels of “Water Expanse.” (A)** Level 1, crossable linear waterway such as a stream; **(B)** Level 2, waterbody is not crossable and viewer can see other side; **(C)** Level 3, waterbody is not crossable and its entire boundary can be seen; **(D)** Level 4, waterbody not crossable and viewer cannot see its other side.

In other words, “water” may be a large predictor, but the form of the water and its landscape layout and/or design is accounting for more of the modeled predictions. This may make sense given that these features have been designed for a purpose, and many times this purpose is to increase aesthetic preference. Knowledge of preferred design form (e.g., the type of form of a semantic feature such as water) can be a powerful tool for design researchers of green and urban spaces. To provide a visual example, Figure 5 shows images and their mean aesthetic preference ratings for each level of the Water Expanse feature. Note that as a single predictor, Water Expanse independently predicts 43.5% of the variance in preference and 44.1% of the variance in naturalness ratings. It is interesting, for example, that image 5D had a high naturalness rating despite having boats in view. This shows the power of the Water Expanse feature in predicting naturalness even if other aspects of the scene would predict otherwise.

Furthermore, in the model for aesthetic preference we note that only two high-level continuous variables remained, suggesting that within the high-level features, the nominal variables containing design and layout information of the scene are carrying the modeled prediction. It is very possible that the general semantic features (continuous variables) are being mediated by the nominal semantic features. Review of changes in the parameter estimates at each level of the nominal variables as independent predictors of aesthetic preference and naturalness provides some insight into how these variables may moderate or mediate relationships between the continuous variables and aesthetic preference and/or naturalness; but a larger study using images that represent a particular range of the high-level nominal variables would be needed to explore that relationship. Such analysis is outside the scope of the current investigation, as there is no particular feature we are interested in; the interest here is in the general relationship of all features. Accordingly, it is also important to note that the features that were excluded from our models may change according to the characteristics of a different set of images. Future studies informed by qualities of a particular feature or theme should investigate those particular interactions and mediations.

Interestingly, when reviewing the nominal variables that remained in the final models, certain levels within these variables stood out as being *more* or *less* preferred/natural, consistent with predictions made by Hunter and Askarinejad (2015). For example, as Table 10 demonstrates, Scenography Types that were bird's eye view of a landscape (level 1) were the most preferred for that feature, and Scenography Types foregrounded with vegetation that extends into the distance (level 5) were least preferred. This result is reminiscent of the savannah hypothesis, which predicts preference for savannah-like places with visibility for survival purposes (Orians and Heerwagen, 1992). For the Building Distribution variable, unsurprisingly, scenes with no buildings (level 0) were most preferred, and those with clusters of buildings were least preferred (levels 2 and 3), which we expect because natural spaces tend to be preferred over urban scenes (Kaplan et al., 1972; Ulrich, 1983; Kardan et al., 2015a; Valtchanov and Ellard, 2015). Similarly, the Built Surfaces for Movement variable demonstrated preference for no built surfaces (level 0) and the least preference for the presence of a network of paths (level 2), which contain various built pathways. Finally, Water Forms containing natural water and natural water boundary edges (level 4) were the most preferred, with more manmade structures being less preferred, and no water (level 0) being least preferred. Not only is this a demonstration of a preference for natural things, but in particular, a specific type of preference for water or perhaps hue.

**Figure 6:**
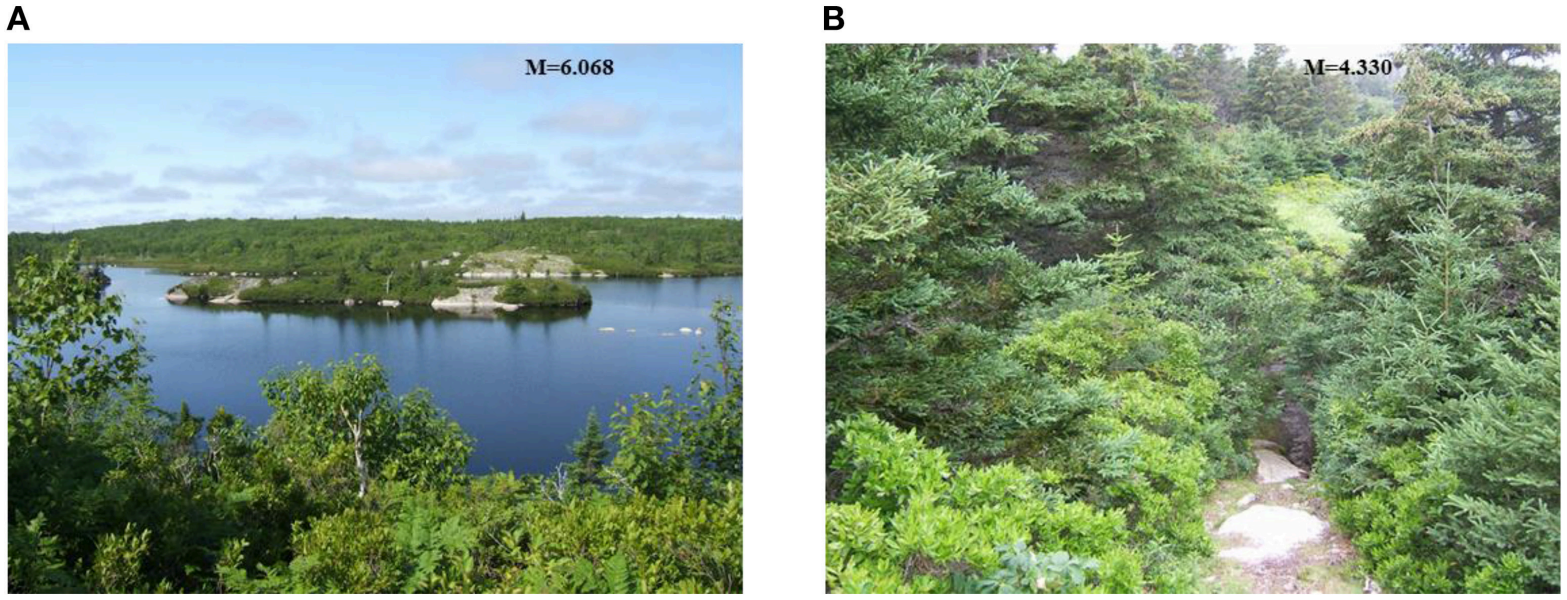
**Comparison of images from the same habitat with different design features, and correspondingly different aesthetic preference ratings**. Mean preference rating for **(A)** and **(B)** are 6.0668 and 4.330, respectively, on a 7-point scale.

As an example of how aesthetic preference for certain levels of the nominal variables may be applied to greenspace design research, we searched the image set to see the types of images with these features (from Table 10) that were more and less preferred. Generally speaking (and without including manmade features, which are all least preferred), the more preferred images are of scenes that contain open views with a natural water body fully in view and a crisp horizon. Conversely, images that are less preferred do not contain water, have sharp skylines, and have views blocked by vegetation; these results are consistent with prospect-refuge theory (Appleton, 1996) which discusses a preference for natural elements that promote survival, such as access to water, and views for finding refuge and avoiding predation. Figure 6 shows, side by side, two images that contain these contrasting features. The images, left to right, have preference ratings of 6.068 and 4.330, respectively, on a 7-point scale. Note Figure 6A contains expansive water but the distant edge is in view, some vegetation, but substantial open space and a generally straight line horizon, compared to Figure 6B which contains low lying vegetation and taller, obstructive trees in the background, and no water. These features are well aligned with the levels that predict more/less aesthetic preference from our analysis, and provide meaningful guidance for greenspace design researchers. Furthermore, knowledge of the specific features that predict aesthetic preference and perceived naturalness can inform future research into applying Attention Restoration Theory, by investigating the relationship between these design features and cognitive restoration effects.

It is important to mention the prominence of the *building* features in this analysis, and their relation to the *vegetation* and *earth* features as those in which humans reside. It is noteworthy that in the final models for aesthetic preference and naturalness, all features dealing with “vegetation” and “earth” fell from the model; furthermore, features dealing with “water,” “sky,” and “buildings” seemed to be carrying the models. It would appear, and certainly in the case of naturalness ratings, that what is important is the presence or absence of buildings and their design orientation, rather than vegetation or earth semantics. Another way to view this interpretation is that for this image set, the absence of buildings entails the presence of vegetation and/or earth. To this end, it isn't the case that vegetation and earth features are unimportant; rather, their importance is encapsulated by the *building* features. This is an important message for future research in green/urban space design; that is, building features (whether measuring the presence or absence of building) are shown in this study to drive the aesthetic preference model as it relates to features in which humans reside.

Additionally, we also speculate about the importance of the presence of water and sky semantic features rather than vegetation, and bring forward this discussion in light of literature by Kellert et al. (2011) that points to preferences people have to green and blue colors, and further delineate this preference with respect to an experimental study by Nutsford et al. (2016). Kellert et al. (2011), and other supporters of biophilia and related fields, discuss the positive feelings and relationship humans have with living systems, and by extension, with associated colors such as green and blue. In this vein, Nutsford et al. (2016) examined the relationship between psychological distress and green and blues spaces. These researchers used geospatial data to quantify these spaces, and then compared those data with health, crime and demographic data of the studied regions. In sum, they found that for a particular city, blue spaces (ocean) were associated with lower psychological distress, while greenspaces were not. Advancing the Nutsford et al. (2016) study, our findings support the importance of blue spaces in predicting aesthetic preference ratings, since *water* and *sky* (blue) features remained in the model and *vegetation* (green) features did not (note that earth semantics were eliminated first, in the analysis of continuous variables, and vegetation semantics were removed afterwards, in the final model of all variables). Relatedly, the hue variable also remained in the aesthetic preference model, demonstrating the importance of this variable. Indeed, as Table 4 shows (linear model using continuous variables to predict aesthetic preference), hue increases in the tabled model (standardized coefficient of 0.216). Since blue is *further* along on a hue color wheel, an interpretation of this standardized coefficient value could be that aesthetic preferences increase as hue becomes more blue. Importantly, our findings should not be misconstrued to diminish the positive effects of greenspaces. For example, numerous studies have found relationships between greenspaces and health and cognitive development outcomes (Mitchell and Popham, 2008; Dadvand et al., 2015; Kardan et al., 2015b); also note that the color green and greenspaces are two different types of features (one low-level, another high-level) and their effects shouldn't be conflated. All we report is support for the role of blue spaces as predictors of aesthetic preference, not that blue or green natural stimuli may foster *more* or *less* positive health outcomes. It could be the case that (as in the case described about *building* features) the *vegetation* features are encapsulated by the *sky/water* features, i.e., more *sky* implies less *vegetation* (*r* = −0.375, *p* < 0.0001). Future studies should explore the relationship between preferences for blue and greenspaces, where those features are experimentally manipulated (and not correlated as in our dataset), as these can have large implications for landscape design research with the aim of enhancing health outcomes.

As Table 11 demonstrates, there are several cases of full mediation, and several more of partial mediation by the high-level features, consistent with a hierarchical perspective on visual processing (e.g., Treisman and Gelade, 1980; Marr, 1982; Biederman, 1987), from which we expected high-level visual features to mediate low-level visual features. Of note, however, is that not all the low-level features are represented in Table 11, and there are also cases of suppression (i.e., low-level visual features relating stronger to aesthetic preference after accounting for mediating high-level semantic features). This suggests that some low-level visual features have stronger direct effects on aesthetic preference than others. For example, for color features we found that the effects of Brightness, Hue, and Saturation on aesthetic preference are mediated by high-level semantic features, but we did not observe such mediation for measures of variance on these features (SD Brightness, SD Hue, and SD Saturation). Regarding edge features, we observed that effects of Straight Edge Density and Disorganized Edge Ratio were mediated by high-level semantic features, but we did not observe mediation for Total Edge Density or Entropy. We speculate that what might be occurring is that some low-level visual features carry more information than others involved in aesthetic judgment; for example, by directly conveying semantic information involved in scene recognition in a parallel process to the hierarchical process (Oliva and Torralba, 2006; Kotabe et al., 2016). Thus, high-level semantic features would explain less of the total effect of these low-level visual features (those that did not make the mediation model) on aesthetic preference, because they are being processed in parallel with the hierarchical process. An interesting direction for future research would be to explore this parallel relationship further, and also identify moderating factors that make a given low-level visual feature have more or less of a direct effect on aesthetic preference. For example, it may depend on the degree to which a low-level visual feature has positive associations with higher-level features such as familiarity, warmth, and safety (Bar and Neta, 2006).

The preceding discussion should not diminish the role of the high-level features as mediators. Brightness, for example, is mediated by eight high-level variables, and when placed with those variables in a multiple mediation model to predict aesthetic preference; the change in *R*^2^ is 0.5037. Furthermore, the only mediations of vegetation features are as suppressors of Brightness. Thus, the effect of Brightness becomes stronger when vegetation features are entered into the model, so in this study there seems to be a certain mediating relationship between vegetation and Brightness, perhaps having to do with a particular shine versus dullness of the vegetation. Indeed, note that Brightness is fully mediated (direct effect = −0.011 [−1.3605, 1.3385]) by the Skyline Vibrancy A variable, which measures canopy-sky interface created by vegetation (a method for measuring vibrancy of vegetation). It is possible that (sufficient) brightness supports the task of deciphering the added information presented by vegetation when reading a scene for resources and safety. This speculation is consistent with aspects of prospect-refuge theory discussed earlier (Appleton, 1996); brightness may help to decipher more details regarding the vegetation and whether it presents a safe or dangerous space. The interesting mediations and suppressions highlighted in this study, such as those between Brightness and Skyline Vibrancy A are a helpful starting point for future studies in the ways low-level features mediate high-level features. We recommend such studies make use of a more specific scene type, where mediators can be more acutely untangled (Hunter and Askarinejad, 2015).

There are a number of limitations in this study. As with other image studies, our interpretations are based on, and limited to interpretations of behavioral responses to static scenes. These results can only inform other research on actual *experiences* of an environment containing these features. However, we would expect that even these scenes present an abstraction of an actual experience in these environments, that there would still be a strong correlation between experiences with these images and experiences with the actual environments that they were derived from. In addition, the analyses captured herein are specific to the image set examined. Although, the image set was carefully selected to represent a mixture of natural and manmade scenes, caution should be taken when applying or extrapolating this analysis to other studies. It would be important to replicate these results using a larger and more varied image set.

These data provide a general picture of the relationship between low- and high- level visual features in predicting preference and naturalness judgments. We have demonstrated that high-level features, and in particular high-level features carrying information about the design content of a scene, are stronger predictors of aesthetic preference and naturalness than the low-level features and much of the variance in predictability of the low-level visual features is mediated by the high-level features. Of course, much of this mediation is driven by the fact that high-level features are composed of low-level visual features. Yet, even with this strength of prediction, some low-level visual features remain independently predictive when taking into account higher-level semantic information. Taken together, by investigating the relationship of low- and high-level features in predicting aesthetic preference and naturalness, designers have a more robust tool set to inform research into landscape design.

## Author contributions

MB developed the study concept and provided supervision throughout. OK and MH collected data for the manuscript. FI conducted data analysis, with assistance from HK and FM, and drafted the manuscript. All authors extensively reviewed and edited the whole manuscript.

### Conflict of interest statement

The authors declare that the research was conducted in the absence of any commercial or financial relationships that could be construed as a potential conflict of interest.
